# Combined interventions for the testing and treatment of HIV and schistosomiasis among fishermen in Malawi: a three-arm, cluster-randomised trial

**DOI:** 10.1016/S2214-109X(24)00283-3

**Published:** 2024-09-18

**Authors:** Augustine T Choko, Kathryn L Dovel, Sekeleghe Kayuni, Donaldson F Conserve, Anthony Buttterworth, Amaya L Bustinduy, J Russell Stothard, Wala Kamchedzera, Madalo Mukoka-Thindwa, James Jafali, Peter MacPherson, Katherine Fielding, Nicola Desmond, Elizabeth L Corbett

**Affiliations:** a Public Health Group, Malawi-Liverpool-Wellcome Trust Clinical Research Programme, Blantyre, Malawi; b Department of International Public Health, Liverpool School of Tropical Medicine, Liverpool, UK; c Department of Tropical Disease Biology, Liverpool School of Tropical Medicine, Liverpool, UK; d Department of Clinical Infection, Microbiology & Immunology, University of Liverpool, Liverpool, UK; e Division of Infectious Diseases, David Geffen School of Medicine, University of California, Los Angeles, CA, USA; f Partners in Hope, Lilongwe, Malawi; g Department of Medicine, Medical Aid Society of Malawi, Blantyre, Malawi; h Department of Prevention and Community Health, George Washington University, Washington, DC, USA; i Department of Clinical Research, London School of Hygiene & Tropical Medicine, London, UK; j Department of Infectious Diseases Epidemiology, London School of Hygiene & Tropical Medicine, London, UK; k School of Health & Wellbeing, University of Glasgow, Glasgow, UK

## Abstract

**Background:**

Undiagnosed HIV and schistosomiasis are highly prevalent among fishermen in the African Great Lakes region. We aimed to evaluate the efficacy of lakeside interventions integrating services for HIV and male genital schistosomiasis on the prevalence of schistosomiasis, uptake of antiretroviral therapy (ART) for HIV, and voluntary male medical circumcision (VMMC) among fishermen in Malawi.

**Methods:**

We conducted a three-arm, cluster-randomised trial in 45 lakeshore fishing communities (clusters) in Mangochi, Malawi. Clusters were defined geographically by their home community as the place where fishermen leave their boats (ie, a landing site). Eligible participants were male fishermen (aged ≥18 years) who resided in a cluster. Clusters were randomly allocated (1:1:1) through computer-generated random numbers to either enhanced standard of care (SOC), which offered invitation with information leaflets to a beach clinic offering HIV testing and referral, and presumptive treatment for schistosomiasis with praziquantel; peer education (PE), in which a nominated fisherman was responsible for explaining the study leaflet to promote services to his boat crew; or peer distribution education (PDE), in which the peer educator explained the leaflet and distributed HIV self-test kits to his boat crew. The beach clinic team and fishermen were not masked to intervention allocation; however, investigators were masked until the final analysis. Coprimary composite outcomes were the proportion of participants who had at least one *Schistosoma haematobium* egg observed on light microscopy from 10 mL of urine filtrate and the proportion who had self-reported initiating ART or scheduling VMMC by day 28. Outcomes were analysed by intention to treat; multiple imputation for missing outcomes was done; random-effect binomial models adjusting for baseline imbalance and clustering were used to compute unadjusted and adjusted risk differences, risk ratios (RRs) and 95% CIs, and intracluster correlation coefficients for each outcome. This trial is registered with ISRCTN, ISRCTN14354324.

**Findings:**

Between March 1, 2022, and Jan 29, 2023, 45 (65·2%) of 69 clusters assessed for eligibility were enrolled in the trial, with 15 clusters per arm. Of the 6036 fishermen screened at baseline, 5207 (86·3%) were eligible for participation: 1745 (87·6%) of 1991 in the enhanced SOC group, 1687 (81·9%) of 2061 in the PE group, and 1775 (89·5%) of 1984 in the PDE group. Compared with the prevalence of active schistosomiasis in the enhanced SOC group (292 [16·7%] of 1745), 241 (13·6%) of 1775 fishermen in the PDE group (adjusted RR 0·80 [95% CI 0·69–0·94]; p=0·0054) and 263 (15·6%) of 1687 fishermen in the PE group (0·92 [0·79–1·07]; p=0·28) had schistosomiasis at day 28. 230 (13·2%) in the enhanced SOC group, 281 (16·7%) in the PE group, and 215 (12·1%) in the PDE group initiated ART or were scheduled for VMMC. ART initiation or VMMC scheduling was not significantly increased with the PDE intervention (0·88 [0·74–1·05); p=0·15) and was marginally increased with the PE intervention (1·16 [0·99–1·37]; p=0·069) when compared with the enhanced SOC group. No serious adverse events were reported in this trial.

**Interpretation:**

We found weak evidence for the use of peer education to increase uptake of ART and VMMC, but strong evidence for the added distribution of HIV self-test kits to promote high engagement with services and reduce the prevalence of active schistosomiasis, suggesting a high potential for scale-up in hard-to-reach communities across Malawi.

**Funding:**

Wellcome Trust and the UK National Institute for Health Research.

## Introduction

There are high burdens of HIV and schistosomiasis in African countries, with an overlap in geographical distribution and affected populations.^1^ Although disease-specific control interventions are in place, potential synergies between these two health hazard control efforts are still being investigated.^2^ A study in Tanzania indicated that, among fishermen, the overall prevalence of HIV in 2021 was 14·2% and schistosome infections was 83·1%.^3^ In Malawi, the prevalence of HIV in 2021 was 8·9%, which is one of the highest in the world and represents 946 000 adults living with HIV, and the incidence rate encompasses 20 000 new infections every year.^4^ Some studies have found an association between having schistosomiasis and an increased susceptibility to HIV infection among women in Zambia,^5^ whereas treatment for schistosomiasis with praziquantel has been shown to improve immune reconstitution on antiretroviral therapy (ART) for HIV in general.^2^

**Research in context**

**Evidence before this study**

We aimed to identify literature on integrated HIV and schistosomiasis services with a focus on Africa. We searched PubMed for titles and abstracts from the date of database inception to Jan 11, 2024, using the following search terms: (HIV) AND (Schistosomiasis). We found 509 records. Overall, we did not find any study that offered combined services for HIV and male genital schistosomiasis targeting fishing communities by the lakeside. Of note, we did not find any study that leveraged the high acceptability of HIV self-testing to increase demand for the uptake of presumptive treatment for male genital schistosomiasis. However, we found evidence suggesting a high prevalence of HIV, male genital schistosomiasis, and other sexually transmitted infections individually and as comorbid conditions in predominantly cross-sectional studies in Africa. Furthermore, a scoping review published in December, 2023, found that, to date, there is no evidence of studies that have integrated service provision with HIV or other sexual health services.

**Added value of this study**

Unlike previous studies that largely simply described opportunities for integrating schistosomiasis and HIV services, to our knowledge, this study is the only large, three-arm, cluster-randomised trial to evaluate the delivery of combined lakeside services for HIV and schistosomiasis among fishing communities in Africa. Additionally, we investigated the innovative use of oral HIV self-test kits, which are popular among men, to increase demand for schistosomiasis treatment. To our knowledge, this was an innovative approach that has not been explored before.

**Implications of all the available evidence**

Our study shows that it is feasible and acceptable to combine services for HIV and schistosomiasis to engage fishing communities at high risk of HIV and schistosomiasis in the African Great Lakes region. We show that offering these services closer to fishing communities by the lakeshore alone increases demand for these services, as evidenced by the large proportion of fishermen engaging in our services in the standard of care group. In this study, we found that offering convenient services might make up for the scarcity of health services offered during the time fishermen are fishing or resting. Our results also show that, by integrating HIV and schistosomiasis services and offering presumptive treatment for schistosomiasis at the same time as an HIV self-test, the uptake of schistosomiasis treatment could be increased. Taken together, these results point to the urgent need to scale up combined services for HIV and schistosomiasis for mobile workers in hard-to-reach communities, such as fishermen.

Fishing communities are far below the UNAIDS target of 95% of people living with HIV knowing their HIV status by 2030, with only around 86% of individuals aware of their HIV-positive status, implying the potential for continued transmission.^6^, ^7^ Studies in Africa have consistently indicated a higher prevalence of HIV among fishing communities than in the general population.^7^ This observation has been partly attributed to high-risk sexual behaviour among these communities, such as so-called fish-for-sex, whereby a large quantity of fish or high-quality fish are exchanged for sex.^8^ Coverage of ART is often lower in fishing communities than in the general population, requiring sustained community-based efforts to increase status knowledge and ART initiation and retention, and to reduce community viral load of HIV.^7^ Additionally, fisherfolk are generally highly mobile and often spend many months away from home, which places them at an increased risk of engaging with high-risk sexual behaviour.^9^

Schistosomiasis is a parasitic infection caused by blood flukes of the genus *Schistosoma*. Infection can result in chronic inflammation of the urinary or gastrointestinal system.^10^ WHO estimates that more than 250 million people worldwide had active schistosomiasis in 2021, 232 million (92·6%) of whom lived in Africa, but only 75·3 million (30·1%) received treatment.^11^ The three main species that infect humans are *Schistosoma haematobium*, *Schistosoma japonicum*, and *Schistosoma mansoni*.^10^ In Malawi, infection with both *S haematobium* and *S mansoni* are common and exposure risk is highly concentrated around bodies of water, especially for fishing communities living in the more densely populated Southern Lakeshore of Lake Malawi.^12^ *S haematobium* affects the genital tract and is referred to as male genital schistosomiasis, whereas *S japonicum* and *S mansoni* affect other parts of the body, including the intestine.^10^

In this study, we aimed to evaluate the efficacy of lakeside interventions integrating services for HIV and male genital schistosomiasis on the prevalence of schistosomiasis, uptake of ART, and voluntary male medical circumcision (VMMC) among fishermen in Malawi.^13^ We hypothesised that the combination of HIV and schistosomiasis services would lead to an improved uptake of testing and treatment for both diseases, resulting in improved management of schistosomiasis and HIV among fishermen in this region.

## Methods

### Study design and participants

We conducted a three-arm, cluster-randomised trial in 45 lakeshore fishing communities across Mangochi, Malawi. Mangochi is a lakeshore district in the eastern region of Malawi and borders Mozambique to the northeast, with an overall population of 1·2 million people in 2018.^14^ In 2016, the prevalence of HIV in Mangochi was 10·1%,^15^ which was above the national average of 8·9%.^4^ The prevalence of schistosomiasis in 2018 was estimated to be 17·1%.^12^

Clusters were defined geographically by their home community as the place on the lakeshore where fishermen leave their boats (ie, a landing site). Between March 8 and Nov 28, 2020, we completed cluster mapping, identifying a total of 69 potential clusters (appendix 1 pp 9–10). Research staff used GPS-enabled tablets to capture the circumference of each cluster and enumerated the number of boats and fishermen in each cluster.

Of the 69 clusters identified, 45 clusters were purposefully selected to ensure a sufficient distance (often ≥1 km) and separation between cluster boundaries to reduce the risk of contamination.^16^ Clusters naturally separated by geographical boundaries, such as rivers, roads, and hills, were prioritised. Enumeration of fishermen was then completed by boat team representatives to establish the number of eligible fishermen for trial participation. Male fishermen aged at least 18 years who were a resident in a cluster (ie, not docking temporarily) were eligible for inclusion in the study.

The detailed study protocol has been described separately.^17^ The protocol refers to outcome measurement at 11 months following trial delivery, which exclusively refers to the total duration of the intervention and not to the period within which outcomes were measured, which was 28 days after trial enrolment.^17^ In these fishing communities, fishing is mainly conducted by a boat crew of around ten men who might be family members, often from the same fishing community. The boat crew is usually made of six to ten people who operate wooden boats, with nightly excursions, and move between lakeshore communities in search of fish.^18^

This trial was approved by the College of Medicine Research Ethics Committee (approval number P.03/20/2975) and the Liverpool School of Tropical Medicine Ethics Committee (approval number 20-027). Consent at the cluster level was obtained from the community leaders with jurisdiction over the clusters. All fishermen provided written informed consent (or a witnessed thumbprint if illiterate) for study participation. Participants, peer educators, and distributors of HIV self-test kits received approximately US$2 for their time; however, individuals who received self-test kits but never returned to the beach clinic did not get this compensation.

### Randomisation and masking

**Figure 1:**
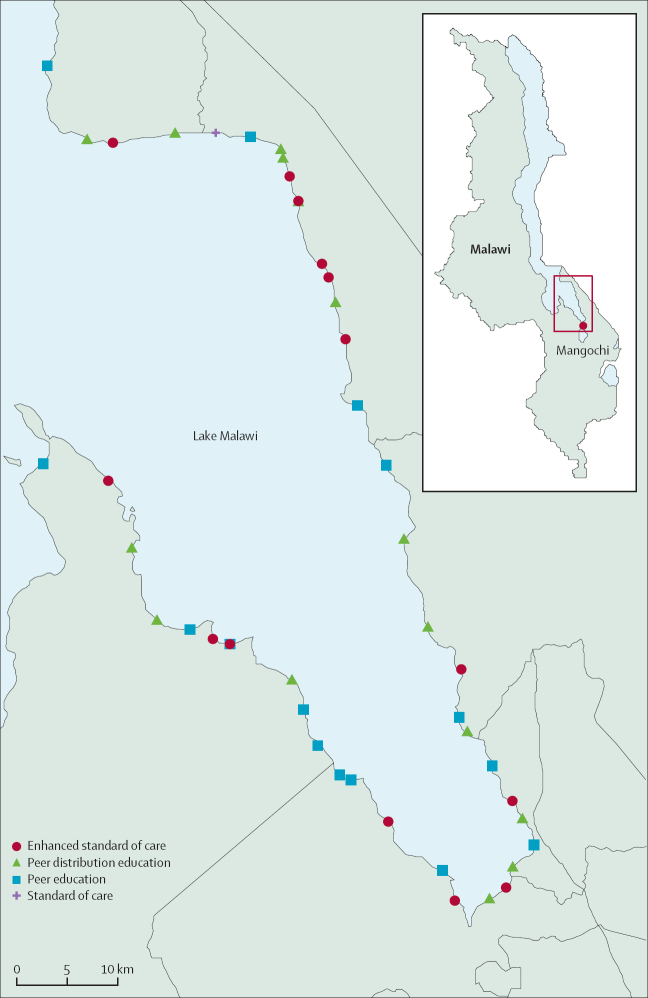
Map of Mangochi and trial clusters Lake Malawi is shown in light blue. Main map boundary lines are provided for Malawi as a country. Reproduced from The Humanitarian Data Exchange.^19^

Clusters were randomly allocated (1:1:1) to either enhanced standard of care (SOC), the peer education (PE) intervention, or the peer distribution education (PDE) intervention (figure 1).^19^ Enhanced SOC involved participants being invited by the beach clinic team with information leaflets to a temporary beach clinic (for only 28 days) offering HIV testing and referral, as well as receiving presumptive treatment for schistosomiasis with praziquantel. The PE intervention comprised a nominated fisherman explaining the study leaflet to promote services to his boat crew. The PDE intervention involved the peer educator not only explaining the leaflet but also distributing HIV self-test kits to his boat crew.

An independent statistician generated the randomisation using computer-generated random numbers. Clusters were allocated to each group at a public meeting after possible allocations were restricted to ensure balance across the three large geographical locations (figure 1). These restriction factors were the number of fishermen per cluster, the known number of fishermen already on ART, and the number of fishermen self-reporting receipt of praziquantel.^20^ The beach clinic team and fishermen were not masked to intervention allocation; however, investigators were masked until the final analysis. The beach clinic teams were responsible for enrolling participants. Representatives of the fishermen were responsible for the assignment to trial groups during a public randomisation ceremony.

### Procedures

Mobile beach clinics were placed at a location chosen by the community leaders on the lakeshore for 28 days in each cluster for all three intervention groups. The beach clinic offered fingerprick HIV testing, confirmatory HIV testing, and referral for ART and VMMC to the nearest health clinic through the study's HIV provider (ie, a certified HIV counsellor responsible for HIV testing, confirmation, and referral). Malawi follows a serial HIV testing algorithm that involves the use of a single rapid test kit with high sensitivity (close to 100%), with a second test done only if the initial test is positive for confirmation and repeat tests offered 2 weeks later for individuals with discordant initial results. Previous arrangement was made with the leadership of the public health facilities closest to the beach clinic. The nearest public health facility from the beach clinic was approximately 1 km away and the farthest was nearly 40 km away, with most beach clinics served by at most two public health facilities. Laboratory microscopists conducted microscopy on urine samples to test for schistosomiasis. Field data collectors completed the endline survey for all fishermen attending the beach clinic for their final visit. Five beach clinics, each staffed with a team comprising an HIV provider, laboratory technicians who conducted microscopy on urine samples to test for schistosomiasis, and data collectors who completed the endline survey for fishermen, covered all 45 clusters over 11 months. A beach clinic was continuously present in a cluster for 28 days, regardless of the intervention group to which they were allocated.

Fishermen heard about the beach clinic through their boat team members, peer educators, or peer distributor–educators. During the participant's first attendance at the beach clinic (day 0), they received a single 40 mg/kg oral dose of presumptive (ie, without being tested) schistosomiasis treatment with praziquantel, regardless of the intervention group to which they had been assigned (appendix 1 pp 9–10). Periodic presumptive treatment of schistosomiasis is not a common approach in many countries, but is supported by both WHO and guidelines in Malawi under mass drug administration protocols for populations at high risk, such as fishermen.^21^ Between day 0 and day 28, serial fingerprick HIV testing was provided to fishermen in the enhanced SOC group and the PE group, and as confirmatory testing in the PDE group. All fishermen who were tested or self-tested and confirmed to have HIV were referred to the nearest public health facility offering ART for initiation and continuation of ART. VMMC scheduling was facilitated for participants who tested negative for HIV but showed interest in the procedure. We designed the study to be neutral in terms of HIV status to minimise stigma and unwanted disclosure*.* We deliberately integrated HIV and schistosomiasis services, so that people in the community or within the study would not be able to identify the type of service a particular participant was seeking from the beach clinic. Additionally, we mixed services so that, even among those participants seeking HIV services, participants would not be able to identify which service another participant was seeking from the beach clinic.

The enhanced SOC group involved access by eligible fishermen to all services offered at the beach clinic, excluding HIV self-test kits. Demand for the beach clinic services in the enhanced SOC group was created by distributing study leaflets to boat teams via a nominated representative of the boat crew. The leaflet included pictorial information about schistosomiasis to enhance accessibility for participants who were illiterate.

In the PE group, a fisherman nominated by his fellow boat crew (one peer educator per boat crew) received training on the services offered at the beach clinic and how to explain the study leaflet to his boat crew. The peer educator received this training over 2 days, which included good clinical practice, the study protocol, and the leaflet, and was responsible for encouraging his peers to use the services at the beach clinic.

The PDE group received the same intervention as the PE group, as well as the distribution of oral HIV self-test kits to the boat crew following an explanation of the study leaflet and the services offered at the beach clinic. Peer educators in the PDE group were called peer distributor–educators, whose roles involved facilitating fishermen's engagement with the beach clinic and training fellow boat crew on how to self-test for HIV. Similarly to peer educators, peer distributor–educators received training on the study leaflet that explained the services available at the beach clinic and linkage to the nearest public health facilities. Additionally, peer distributor–educators received instructions for the use of HIV self-test kits and a demonstration with a cotton wool bud on how to self-test correctly. Each peer distributor–educator received up to 12 oral HIV self-test kits. Although peer distributor–educators provided access to HIV self-test kits and the beach clinic, the design did not require their boat crew to disclose their self-test result to them. However, all fishermen receiving HIV self-test kits were strongly encouraged to seek confirmatory HIV testing at the beach clinic in case of a positive result.

On day 28, the final study visit, fishermen who voluntarily returned to the beach clinic and presented the study coupon completed a face-to-face questionnaire on tablets running an open data kit. Furthermore, these participants provided a urine sample for an egg count to be performed. The study microscopist conducted an egg count per 10 mL of urine using filtration of a single urine specimen through Nuclepore membranes (Whatman International, Maidstone, UK).^9^ For quality assurance, 10% re-reading of positive and negative slides was done by a masked second reader from Mangochi District Hospital. Participants with active schistosomiasis were provided with a new oral dose of 40 mg/kg praziquantel.

### Outcomes

All trial outcomes were measured at 28 days after enrolment. The first coprimary composite outcome was the proportion of participants who had at least one *S haematobium* egg observed on light microscopy from 10 mL of urine filtrate (egg-positive) in each intervention group. The second coprimary composite outcome was the proportion of fishermen who self-reported starting ART or scheduling VMMC, both of which were confirmed by verifying with the ART register or by verifying with the VMMC team. The composite outcome of initiating ART or scheduling VMMC was chosen because these are the two most effective interventions for the treatment and prevention of schistosomiasis and HIV available in this setting; if someone tests negative for HIV, they should sign up for VMMC, and if they test positive for HIV, they should start ART. Under randomisation, all intervention groups would be assumed to be the same, except for one group that receives the intervention while the other does not. Thus, the main comparisons were based on follow-up estimates at 28 days, rather than baseline estimates. Microscopy was done after 4 weeks to ensure the maximum effect of praziquantel in killing all adult worms. The ART register is a paper-based document that holds a record for individuals who start treatment for HIV. Patient confidentiality was maintained by use of the study number, and not names, to link the ART register record and study through the study's HIV provider.

Secondary outcomes were the proportion of participants testing for HIV, measured by the number of fishermen presenting for testing at the beach clinic in the enhanced SOC and the PE groups and reporting the HIV self-test in the PDE group; the proportion of participants who indicated that they approved the use of pre-exposure prophylaxis (PrEP) for preventing HIV transmission as an indicator of HIV prevention knowledge; the geometric mean number and SD of eggs per 10 mL of urine filtrate in each intervention group; the proportion of participants self-reporting high-risk sexual behaviour in the previous month; and the proportion of participants showing knowledge of schistosomiasis, which was assessed through a questionnaire. The costs of beach clinic services and unit costs per intervention group were also measured (appendix 1 pp 6–9).

### Statistical analysis

For the first coprimary composite outcome, we assumed praziquantel uptake would be 10–20% higher in the PDE and PE groups than the conservatively assumed rate of 40–60% in the enhanced SOC group, with a baseline egg positivity rate of 15–25%,^12^ 95% cure from praziquantel, and a *k* value of 0·20–0·30.^16^ Power provided by the 15 clusters per arm for HIV endpoints was more than 80% over most of this range of scenarios. There was no sample size adjustment for multiplicity of testing (statistical testing of more than one null hypothesis), although a 10% adjustment for loss to follow-up with missing outcomes was made.

For the second coprimary composite outcome, we assumed that 10% of participants in the enhanced SOC group would initiate ART or schedule VMMC, and that the PDE intervention could increase this estimate to 19%.^22^ With an assumed harmonic mean cluster size of 100 fishermen per cluster^20^ and a coefficient of variation of 0·20,^23^ 15 clusters per arm (1500 fishermen per arm) provided 80% power with an alpha level of 0·05 to detect at least a difference this large.

All outcomes were analysed in R (version 4.3.1) following intention-to-treat principles, with multiple imputation performed with the miceadds R package^24^ for participants with missing outcome data at day 28. A prespecified modified intention-to-treat analysis was performed for the coprimary composite outcome of ART initiation or scheduled VMMC by excluding the number of participants already on ART from the denominator. We followed CONSORT guidelines in reporting preplanned study outcomes (appendix 1 pp 1–5).^25^ We used random-effect binomial regression models to compute the unadjusted and adjusted risk differences, risk ratios (RRs) and 95% CIs, and intracluster correlation coefficients for each outcome. We computed estimates for the PE and the PDE groups using the enhanced SOC group as the comparator. In each model, we adjusted for clustering using the identification number of the cluster and for baseline imbalance on literacy, circumcision status, and HIV testing history. p values of up to and including 0·05 were considered to be statistically significant.

We conducted a partial cost analysis of the PE and the PDE interventions, with a focus on schistosomiasis testing and treatment, HIV testing, and either ART initiation or scheduled VMMC from a programmatic perspective (appendix 1 pp 6–7).^26^ We did not include the cost of care provision related to HIV or VMMC services at the public health facility to which the participants were referred to after receiving care at our beach clinic. Additionally, we performed a cost-effectiveness analysis of the interventions (appendix 1 pp 6–7). There is no formal cost-effectiveness threshold in Malawi regarding this study's outcomes. To the best of our knowledge, there have not been similar trials with economic evaluation studies conducted within a similar population. Therefore, this study only reports incremental cost-effectiveness ratios without relation to a threshold of willingness to pay. To test the robustness of our results, we conducted a probabilistic sensitivity analysis, in which we varied the total costs and outcomes per arm (appendix 1 pp 6–7).^27^

Adverse events were monitored through independent reporting systems by leaders of the beach clinic teams and anonymous reporting by fishermen. However, we aimed to report serious adverse events (grade 3 or 4) only because all grade 1 and 2 events were within national standard testing and treatment guidelines in Malawi and were not required to be reported by the institutional review boards that reviewed the study. This trial is registered with ISRCTN, ISRCTN14354324.

### Role of the funding source

The funders of the study had no role in study design, data collection, data analysis, data interpretation, or writing of the report.

## Results

**Figure 2:**
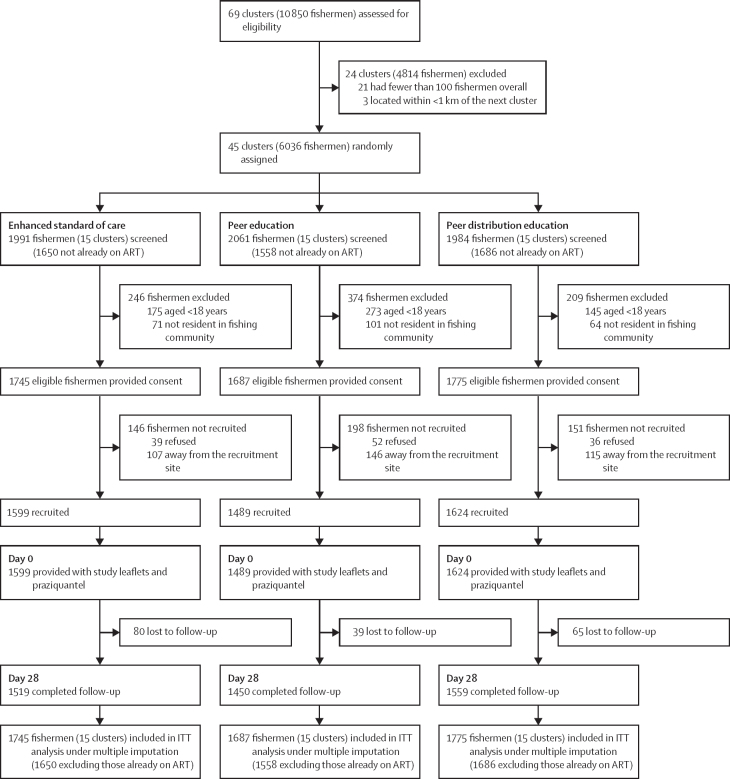
Trial profile ART=antiretroviral therapy. ITT=intention-to-treat.

Between March 1, 2022, and Jan 29, 2023, 45 (65·2%) of 69 clusters assessed for eligibility were enumerated and enrolled in the trial, with 15 clusters per arm. Clusters varied in size, with a median of 135 fishermen per cluster (IQR 98–200). Of the 6036 fishermen screened at baseline, 5207 (86·3%) were eligible for participation: 1745 (87·6%) of 1991 in the enhanced SOC group, 1687 (81·9%) of 2061 in the PE group, and 1775 (89·5%) of 1984 in the PDE group (figure 2). A total of 1519 (87·0%) fishermen in the enhanced SOC group, 1450 (86·0%) in the PE group, and 1559 (87·8%) in the PDE group had their outcome status ascertained at day 28. Baseline characteristics were reasonably balanced, except for literacy, circumcision status, and HIV testing history (table 1). Overall, mean age was 33 years (SD 12), 2171 (41·7%) fishermen could not read or write, 308 (5·9%) were taking ART, and 2290 (44·0%) of 5207 had active schistosomiasis at baseline (95% CI 0·24–0·65), with a geometric mean of 7·3 eggs per 10 mL of urine (95% CI 3·2–16·8). 776 (14·9%) fishermen reported undergoing medical circumcision, with the majority of circumcisions being traditional (3377 [64·9%]), and 2415 (65·6%) fishermen had not taken praziquantel in the previous 12 months. No serious adverse events were observed or reported throughout the study period.

**Table 1:** Baseline characteristics

		**Enhanced standard of care (n=1745)**	**Peer education (n=1687)**	**Peer distribution education (n=1775)**	**Overall (n=5207)**
Age, years		33·20 (12·49)	33·81 (12·06)	32·61 (12·43)	33·20 (12·34)
	Data missing	1 (0·1%)	0	1 (0·1%)	2 (<1·0%)
Able to read and write?					
	No	812 (46·5%)	603 (35·7%)	756 (42·6%)	2171 (41·7%)
	Yes	932 (53·4%)	1082 (64·1%)	1018 (57·4%)	3032 (58·2%)
	Data missing	1 (0·1%)	2 (0·1%)	1 (0·1%)	4 (<1·0%)
Highest level of education attained					
	None	532 (30·5%)	342 (20·3%)	505 (28·5%)	1379 (26·5%)
	Primary	904 (51·8%)	973 (57·7%)	953 (53·7%)	2830 (54·3%)
	Secondary, no MSCE	245 (14·0%)	295 (17·5%)	251 (14·1%)	791 (15·2%)
	Secondary, MSCE	52 (3·0%)	68 (4·0%)	55 (3·1%)	175 (3·4%)
	Higher	12 (0·7%)	8 (0·5%)	9 (0·5%)	29 (0·6%)
	Data missing	0	1 (0·1%)	2 (0·1%)	3 (<1·0%)
Marital status					
	Divorced, separated, or widowed	141 (8·1%)	182 (10·8%)	113 (6·4%)	436 (8·4%)
	Never married	452 (25·9%)	373 (22·1%)	535 (30·1%)	1360 (26·1%)
	Married	1150 (65·9%)	1130 (67·0%)	1121 (63·2%)	3401 (65·3%)
	Data missing	2 (0·1%)	2 (0·1%)	6 (0·3%)	10 (<1·0%)
Ever tested for HIV?					
	No	327 (18·7%)	293 (17·4%)	371 (20·9%)	991 (19·0%)
	Yes	1417 (81·2%)	1393 (82·6%)	1403 (79·0%)	4213 (80·9%)
	Data missing	1 (0·1%)	1 (0·1%)	1 (0·1%)	3 (<1·0%)
Tested for HIV in last 12 months?					
	No	545 (38·5%)	541 (38·9%)	601 (42·8%)	1687 (40·1%)
	Yes	872 (61·5%)	850 (61·1%)	802 (57·2%)	2524 (59·9%)
	Data missing	0	2 (0·1%)	0	2 (<1·0%)
Currently on ART?					
	No	1650 (94·6%)	1558 (92·4%)	1686 (95·0%)	4894 (94·0%)
	Yes	95 (5·4%)	126 (7·5%)	87 (4·9%)	308 (5·9%)
	Data missing	0	3 (0·2%)	2 (0·1%)	5 (<1·0%)
Circumcision status					
	Not circumcised	372 (21·3%)	391 (23·2%)	287 (16·2%)	1050 (20·2%)
	Medically circumcised	244 (14·0%)	271 (16·1%)	261 (14·7%)	776 (14·9%)
	Traditionally circumcised	1128 (64·6%)	1024 (60·7%)	1225 (69·0%)	3377 (64·9%)
	Data missing	1 (0·1%)	1 (0·1%)	2 (0·1%)	4 (<1·0%)
Ever taken praziquantel?					
	No	520 (29·8%)	481 (28·5%)	503 (28·3%)	1504 (28·9%)
	Yes	1224 (70·1%)	1205 (71·4%)	1264 (71·2%)	3693 (71·1%)
	Data missing	1 (0·1%)	1 (0·1%)	8 (0·5%)	10 (<1·0%)
Taken praziquantel in last 12 months?					
	No	799 (65·5%)	770 (64·0%)	846 (67·1%)	2415 (65·6%)
	Yes	421 (34·5%)	433 (36·0%)	415 (32·9%)	1269 (34·4%)
	Data missing	4 (0·3%)	2 (0·2%)	3 (0·2%)	9 (<1·0%)
Know anyone who died of HIV?					
	No	868 (53·9%)	878 (52·5%)	830 (50·4%)	2576 (52·3%)
	Yes	741 (46·1%)	794 (47·5%)	818 (49·6%)	2353 (47·7%)
	Data missing	136 (7·8%)	15 (0·9%)	127 (7·2%)	278 (5·3%)
Self-rated general health					
	Excellent	362 (20·7%)	351 (20·8%)	446 (25·1%)	1159 (22·3%)
	Good	1121 (64·2%)	1012 (60·0%)	1097 (61·8%)	3230 (62·0%)
	Fair	144 (8·3%)	165 (9·8%)	156 (8·8%)	465 (8·9%)
	Poor	118 (6·8%)	159 (9·4%)	75 (4·2%)	352 (6·8%)
	Data missing	0	0	1 (0·1%)	1 (<1·0%)
Active schistosomiasis at baseline*		0·44 (0·24–0·65)	0·44 (0·24–0·65)	0·44 (0·24–0·65)	0·44 (0·24–0·65)

Data are mean (SD), n (%), or proportion (95% CI). ART=antiretroviral therapy. MSCE=Malawi School Certificate of Education.

* Estimate of participants with at least one egg in 10 mL of urine filtrate. The estimate is the same because results were not stratified by arm at baseline to avoid the ethical dilemma of having to immediately treat individuals with schistosomiasis and potentially bias the results and trial conduct.

There was a significant reduction in the proportion of fishermen with active schistosomiasis in the PDE group compared with the enhanced SOC group (241 [13·6%] of 1775 *vs* 292 [16·7%] of 1745; table 2). The unadjusted RR for this comparison was 0·81 (95% CI 0·69–0·95; p=0·0087) and remained significant after adjustment (adjusted RR 0·80 [0·69–0·94]; p=0·0054). However, there was no significant difference in the prevalence of active schistosomiasis between the enhanced SOC group and the PE group (unadjusted RR 0·93 [0·80–1·09]; p=0·37). This finding did not change after adjustment (adjusted RR 0·92 [0·79–1·07]; p=0·28).

**Table 2:** Primary and secondary outcomes

		**Enhanced standard of care (n=1745)**	**Peer education (n=1687)**	**Peer distribution education (n=1775)**
**Primary outcomes**				
Fishermen with active schistosomiasis*		292 (16·7%)	263 (15·6%)	241 (13·6%)
	Risk difference (95% CI)	..	−1·13 (−3·59 to 1·32)	−3·15 (−5·5 to −0·81)
	Unadjusted RR (95% CI); p value	1 (ref)	0·93 (0·80 to 1·09); 0·37	0·81 (0·69 to 0·95); 0·0087
	Adjusted RR† (95% CI); p value	1 (ref)	0·92 (0·79 to 1·07); 0·28	0·80 (0·69 to 0·94); 0·0054
Fishermen initiated ART or scheduled for VMMC during study period‡		230 (13·2%)	281 (16·7%)	215 (12·1%)
	Risk difference (95% CI)	..	3·44 (1·01 to 5·86)	−1·06 (−3·32 to 1·19)
	Unadjusted RR (95% CI); p value	1 (ref)	1·26 (1·07 to 1·48); 0·0057	0·92 (0·77 to 1·10); 0·36
	Adjusted RR† (95% CI); p value	1 (ref)	1·16 (0·99 to 1·37); 0·069	0·88 (0·74 to 1·05); 0·15
Fishermen tested for HIV‡		1406/1650 (85·2%)	1315/1558 (84·4%)	1483/1686 (88·0%)
	Risk difference (95% CI)	..	0·10 (−1·00 to 0·10)	3·00 (2·00 to 4·00)
	Unadjusted RR (95% CI); p value	1 (ref)	1·00 (0·98 to 1·02); 0·75	1·03 (1·01 to 1·05); 0·011
	Adjusted RR† (95% CI); p value	1 (ref)	1·00 (0·99 to 1·01); 0·53	1·01 (1·01 to 1·02); 0·0087
**Secondary outcomes**				
Fishermen perceived acceptability of pre-exposure prophylaxis		1130 (64·8%)	1329 (78·8%)	1238 (69·7%)
	Risk difference	..	13·8 (10·77 to 16·82)	4·67 (1·51 to 7·83)
	Unadjusted RR (95% CI); p value	1 (ref)	1·22 (1·17 to 1·27); <0·0001	1·08 (1·03 to 1·13); 0·0016
	Adjusted RR† (95% CI); p value	1 (ref)	1·22 (1·17 to 1·27); <0·0001	1·08 (1·03 to 1·13); <0·0001
Self-reported high-risk sexual behaviour in the past 1 month		96 (5·5%)	124 (7·4%)	69 (3·9%)
	Risk difference (95% CI)	..	1·85 (0·21 to 3·49)	−1·61 (−3·01 to −0·22)
	Unadjusted RR (95% CI); p value	1 (ref)	1·34 (1·04 to 1·73); 0·024	0·71 (0·52 to 0·95); 0·024
	Adjusted RR† (95% CI); p value	1 (ref)	1·29 (1·00 to 1·67); 0·049	0·72 (0·54 to 0·97); 0·033
Self-reported schistosomiasis knowledge		1612 (92·4%)	1568 (92·9%)	1607 (90·5%)
	Risk difference (95% CI)	..	0·67 (−1·10 to 2·44)	−1·85 (−3·74 to 0·03)
	Unadjusted RR (95% CI); p value	1 (ref)	1·01 (0·99 to 1·03); 0·45	0·98 (0·96 to 1·00); 0·051
	Adjusted RR† (95% CI); p value	1 (ref)	1·00 (0·98 to 1·02); 0·86	0·98 (0·96 to 1·00); 0·015

Data are n (%), unless otherwise specified. RR=risk ratio. ART=antiretroviral therapy. VMMC=voluntary male medical circumcision.

* Measured within 28 days, defined as at least one *Schistosoma haematobium* egg seen on light microscopy of the filtrate from 10 mL of urine (egg-positive).

† Adjusted for clustering, literacy, and HIV testing in previous 12 months and VMMC status. Intracluster correlation coefficient was 0·08, estimated from the adjusted model.

‡ Excludes individuals already on ART.

**Figure 3:**
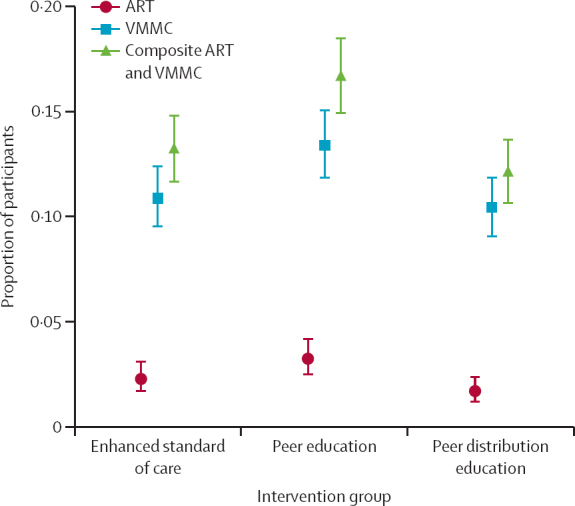
Proportion of participants who had initiated ART or scheduled VMMC by day 28 Error bars indicate binomial exact lower and upper confidence limits for the proportion estimate. ART=antiretroviral therapy. VMMC=voluntary male medical circumcision.

230 (13·2%) fishermen in the enhanced SOC group, 281 (16·7%) in the PE group, and 215 (12·1%) in the PDE group initiated ART or were scheduled for VMMC during the study period (table 2, figure 3). The PDE intervention did not significantly increase the proportion of fishermen initiating ART or scheduling VMMC in the unadjusted analysis (RR 0·92 [95% CI 0·77–1·10]; p=0·36) or the adjusted analysis (adjusted RR 0·88 [0·74–1·05]; p=0·15; figure 3). The PE intervention significantly increased the proportion of participants initiating ART or scheduling VMMC in the unadjusted analysis (RR 1·26 [1·07–1·48]; p=0·0057); however, only marginal evidence of an increase was observed in the adjusted analysis (adjusted RR 1·16 [0·99–1·37]; p=0·069).

HIV testing was similar between the enhanced SOC group and the PE group, and did not differ between the unadjusted analysis and the adjusted analysis (table 2). The addition of oral HIV self-test kits significantly increased the proportion of fishermen testing for HIV in the PDE group compared with the enhanced SOC group. This finding was significant in the unadjusted analysis and remained significant in the adjusted analysis (table 2).

Fishermen were asked: “Do you approve of people who take a pill every day to prevent getting HIV?” at day 28. Compared with the enhanced SOC group, the proportion of participants who approved hypothetical PrEP use (responding yes to the above question) was significantly higher in both the PE group (unadjusted RR 1·22 [95% CI 1·17–1·27); p<0·0001 and adjusted RR 1·22 [1·17–1·27]; p<0·0001) and the PDE group (unadjusted RR 1·08 [1·03–1·13]; p<0·0001 and adjusted RR 1·08 [1·03–1·13]; p<0·0001).

In terms of intensity of schistosomiasis infection, among the 600 (11·5%) fishermen who still had at least one egg observed in 10 mL of urine filtrate at day 28, the overall geometric mean number of eggs was 6·33 eggs per 10 mL of urine filtrate (95% CI 5·60–7·15). Intensity of infection was similar between the enhanced SOC group (geometric mean 5·55 eggs [95% CI 4·50–6·84]), the PE group (6·82 [5·51–8·45]), and the PDE group (6·72 [5·44–8·30]).

Compared with the enhanced SOC group, the proportion of participants self-reporting high-risk sexual behaviour in the previous 1 month was significantly higher in the PE group but significantly lower in the PDE group. Self-reported knowledge of schistosomiasis was high overall, with similar estimates between participants in the enhanced SOC group and the PE group; however, this knowledge was marginally lower in the PDE group than in the enhanced SOC group (adjusted RR 0·98 [95% CI 0·96–1·00]; p=0·015).

In the complete-case analysis (performed as a sensitivity analysis), we observed minor changes in the results (appendix 1 pp 16–17), especially around the two coprimary outcomes. The larger sample size under multiple imputation offered more statistical power to detect the differences in outcomes. For example, in the complete-case analysis, there was no significant reduction in the proportion of fishermen with active schistosomiasis in the PDE group compared with the enhanced SOC group, contrary to what has been observed under multiple imputation (table 2). However, there was strong evidence in the complete-case analysis that the PE intervention increased the proportion of fishermen initiating ART or scheduling VMMC (appendix 1 pp 16–17), which was simply marginal under multiple imputation (table 2).

The total cost of delivering all services at the beach clinic was the lowest in the PDE group at US$26 944·03, compared with $27 864·62 in the enhanced SOC group and $27 158·73 in the PE group (appendix 1 p 12). The average cost per fisherman tested for HIV was the lowest in the PDE group at $4·39 and highest in the PE group at $5·76 (appendix 1 p 12), whereas the cost per fisherman linked to ART initiation or VMMC scheduling was lowest in the PE group at $1·61, compared with $1·70 in the enhanced SOC group and $1·73 in the PDE group. The cost per fisherman treated for schistosomiasis was $5·04 in the enhanced SOC group, $5·13 in the PE group, and $4·97 in the PDE group. Compared with the enhanced SOC group, the incremental cost per additional fisherman treated for schistosomiasis in the PE group was $11·42. Given that treating schistosomiasis was less costly and more effective with the PDE intervention than with enhanced SOC, the PDE group was dominant (appendix 1 pp 13–14). Conducting HIV testing under the PE group was dominated by the enhanced SOC group; however, the PDE group was dominant (appendix 1 pp 13–14). For the composite outcome of ART initiation or VMMC scheduling, the incremental cost per additional fisherman for linkage to these services was $3·76 in the PE group and $73·84 in the PDE group. The results of the probabilistic sensitivity analysis showed similar findings (appendix 1 p 15).

## Discussion

In this three-arm, cluster-randomised trial, we found that mobile beach clinics targeting fishermen in Mangochi, Malawi, were largely successful at increasing the uptake of services for the treatment of schistosomiasis and HIV among this community. To our knowledge, this is the only cluster-randomised trial to date that has examined the effect of integrated services for the treatment of HIV and schistosomiasis, including HIV self-testing, ART, VMMC, and schistosomiasis treatment with praziquantel. Linkage to additional services (ie, ART initiation or VMMC scheduling) was higher in the PE group than in the enhanced SOC group, but there was no difference between the PDE group and the enhanced SOC group. The PDE intervention was associated with a significant reduction in the prevalence of active schistosomiasis compared with enhanced SOC. HIV testing was high across all intervention groups, including the enhanced SOC group (78–84% of fisherman tested for HIV). Hypothetical acceptability of PrEP was generally high across all intervention groups, with slightly higher acceptance in the PE group than in the PDE and enhanced SOC groups.

We found that beach clinics offering peer education and distribution of HIV self-test kits increased coverage of HIV testing among fishermen in Malawi. There is increasing literature showing that men care about their own health (and the health of others) and desire access to health services.^28^ However, services based at health facilities are often not accessible for men with informal employment, such as fishermen, because attending a health facility often means forfeiting a day's wage. Providing services at beach clinics seems to overcome numerous barriers to seeking care for HIV and schistosomiasis by directly targeting fishermen near their workplace and providing private services at flexible hours. Similar findings have been found throughout the region.^28^ Workplace services can benefit individuals who are unable to attend health facilities by increasing convenience and reducing the need to take time off to seek care. By extension, these beach clinics might offer a practical mechanism for delivering other equally important services, such as screening and treatment for sexually transmitted infections and testing and treatment for malaria, for communities who are hard to reach in the African region. Future studies should consider innovative ways to provide workplace health services for informal mobile workers.

The addition of peer education slightly improved ART initiation and VMMC scheduling at the nearest public health facilities compared with informational leaflets alone, but the distribution of HIV self-test kits did not improve participants’ use of HIV treatment or prevention services. Peer education might improve ART initiation and VMMC scheduling due to peer support and peer-based motivation. Other studies show that men respect and value peer interactions when talking about health services.^28^ The limited effect of peer education when combined with HIV self-testing on linkage to treatment or prevention could indicate remaining barriers to accessing additional services after use of HIV self-test kits. There is mixed evidence on linkage to treatment after self-testing HIV positive.^28^ One study in Malawi found that men who received a positive HIV self-test result after testing alone (without a health-care worker present) desired peer mentorship and counselling to support linkage to care.^29^ Another perspective is that HIV self-testing often results in individuals learning about a positive HIV status while otherwise healthy; therefore, it can take longer than the 11 month study period for them to experience symptoms and engage in treatment.^27^

There was strong evidence of an association between the distribution of HIV self-test kits and a reduced prevalence of active schistosomiasis. This seemingly unusual finding might be explained by the high coverage of HIV self-test kit distribution and the participants’ engagement with the beach clinics and HIV testing in the PDE group. For example, we previously found that fishermen who reported previous HIV testing in this same setting were more likely to report a history of taking praziquantel.^20^ Thus, the high HIV testing in the PDE group might indicate a high acceptability of taking praziquantel compared with the enhanced SOC group.

This trial has several limitations. First, we were not able to measure actual VMMC uptake. The study participants were only scheduled for a VMMC appointment, but we are unable to confirm if VMMC was actually performed in each case following scheduling. Second, we did not include a real-world standard of care group, given that beach clinics are not routinely implemented as part of Malawi's Ministry of Health services. Our findings show that just having an outreach clinic on the beach, with or without peer educators, increases fishermen's access to services for both schistosomiasis and HIV. Additional research is needed to compare the efficacy of services delivered by beach clinics with true standard of care. Third, ART initiation and VMMC scheduling were assessed at 28 days after trial enrolment. Individuals might have engaged in treatment or preventive services after the 28-day period, potentially underestimating the effects of interventions. Underestimates of ART or VMMC engagement would probably affect the PDE group the most because clients using HIV self-test kits might take longer to link to additional services than those offered enhanced SOC. Fourth, the study had enough power to detect the significant difference in the composite outcome of ART initiation or VMMC scheduling between enhanced SOC and the PE intervention. However, the study was not powered to detect differences in retention on HIV treatment or HIV viral suppression, which are important outcomes for individual and community health.

Due to the short follow-up period of 28 days, use of quality-adjusted life-years as health outcomes for the cost-effectiveness analysis might not have been appropriate. Therefore, the comparability of our findings to other diseases and their respective interventions is limited. Consequently, our findings might not be comparable to studies with longer follow-up periods. This consideration is crucial for resource-constrained settings, such as Malawi, because decisions need to be made on where best to allocate already limited resources. Additionally, in the absence of a viable willingness-to-pay threshold in Malawi for the study's outcomes, we could not make strong conclusions on whether the interventions would be cost-effective for Malawi's health system.

Cost-efficient and integrated services for mobile individuals with informal occupations, such as fishermen, are needed. Beach clinics offering informative leaflets, peer education alone, or peer education combined with the distribution of HIV self-test kits showed a promising effect on the engagement of fishermen with services in Malawi. Additional research is needed to assess how to support ongoing ART engagement, VMMC completion, and schistosomiasis treatment in this community in Malawi.

### Contributors

### Equitable partnership declaration

### Data sharing

Individual de-identified participant data (including data dictionaries) will be made publicly available through the London School of Hygiene & Tropical Medicine data repository, including baseline data and data on primary and secondary outcomes. Data will become permanently available upon publication and will be openly accessible to anyone who wishes to perform an analysis. A link to the data will be provided.

## Declaration of interests

ELC reports grants to London School of Hygiene & Tropical Medicine during the conduct of the study. All other authors declare no competing interests. DFC was supported by grants from the National Institutes of Health (grant number R00MH110343: PI: DFC and R01NR021169: PI: DFC).

## Supplementary Materials

Supplementary appendix 1

Equitable Partnership Declaration
