# Supplementary material for: Combined interventions for the testing and treatment of HIV and schistosomiasis among fishermen in Malawi: a three-arm, cluster-randomised trial
Source: Lancet Glob Health. 2024 Sep 18;12(10):e1673–83. doi: 10.1016/S2214-109X(24)00283-3 (PMC11420466; doi:10.1016/S2214-109X(24)00283-3)
Supplement: Supplementary appendix 1 [file mmc1.pdf]

THE LANCET
Global Health

## Supplementary appendix 1

This appendix formed part of the original submission and has been peer reviewed. We post it as supplied by the authors.

Supplement to: Choko AT, Dovel KL, Kayuni S, et al. Combined interventions for the testing and treatment of HIV and schistosomiasis among fishermen in Malawi: a three-arm, cluster-randomised trial. *Lancet Glob Health* 2024; **12:** e1673–83.

# Contents

APPENDIX: Methods 1: CONSORT Checklist ........ 1

APPENDIX: Methods 2: Costing methods ........ 5

APPENDIX: Table 1: Unit costs of healthcare resource use items ........ 7

APPENDIX: Figure 1: Timing of trial activities and follow up ........ 10

APPENDIX: Table 2: Total costs by trial arm (2022 USD$) ........ 10

APPENDIX: Table 3: Cost per fisherman by trial arm (2022 USD$) ........ 10

APPENDIX: Table 4: Incremental cost effectiveness ratios ........ 11

APPENDIX: Figure 2: Cost-effectiveness scatter plots for sensitivity analysis ........ 13

APPENDIX: Table 5: Primary and secondary outcomes under complete case (sensitivity analysis) ........ 13

APPENDIX: Table 6: Primary and secondary outcomes results using logistic regression ........ 15

# APPENDIX: Methods 1: CONSORT Checklist

| Section/Topic | Item No | Standard Checklist item | Extension for cluster designs | Page No * |
|---|---|---|---|---|
| **Title and abstract** | | | | |
| | 1a | Identification as a randomised trial in the title | Identification as a cluster randomised trial in the title | 1 |
| | 1b | Structured summary of trial design, methods, results, and conclusions (for specific guidance see CONSORT for abstracts)[i,ii] | See table 2 | 3-4 |
| **Introduction** | | | | |
| **Background and objectives** | 2a | Scientific background and explanation of rationale | Rationale for using a cluster design | 6 |
| | 2b | Specific objectives or hypotheses | Whether objectives pertain to the cluster level, the individual participant level or both | 6 |
| **Methods** | | | | |
| **Trial design** | 3a | Description of trial design (such as parallel, factorial) including allocation ratio | Definition of cluster and description of how the design features apply to the clusters | 6-7 |
| | 3b | Important changes to methods after trial commencement (such as eligibility criteria), with reasons | | **N/A** |
| **Participants** | 4a | Eligibility criteria for participants | Eligibility criteria for clusters | 7 |
| | 4b | Settings and locations where the data were collected | | **7** |
| **Interventions** | 5 | The interventions for each group with sufficient details to allow replication, including how and when they | Whether interventions pertain to the cluster level, the individual participant level or both | 8-9 |

| | | | | |
|---|---|---|---|---|
| | | were actually administered | | |
| **Outcomes** | 6a | Completely defined pre-specified primary and secondary outcome measures, including how and when they were assessed | Whether outcome measures pertain to the cluster level, the individual participant level or both | 9-10 |
| | 6b | Any changes to trial outcomes after the trial commenced, with reasons | | **N/A** |
| **Sample size** | 7a | How sample size was determined | Method of calculation, number of clusters(s) (and whether equal or unequal cluster sizes are assumed), cluster size, a coefficient of intracluster correlation (ICC or *k*), and an indication of its uncertainty | 10-11 |
| | 7b | When applicable, explanation of any interim analyses and stopping guidelines | | **N/A** |
| **Randomisation:** | | | | |
| **Sequence generation** | 8a | Method used to generate the random allocation sequence | | **11** |
| | 8b | Type of randomisation; details of any restriction (such as blocking and block size) | Details of stratification or matching if used | 11 |
| **Allocation concealment mechanism** | 9 | Mechanism used to implement the random allocation sequence (such as sequentially numbered containers), describing any steps taken to conceal the sequence until interventions were assigned | Specification that allocation was based on clusters rather than individuals and whether allocation concealment (if any) was at the cluster level, the individual participant level or both | 11 |

| | | | | |
|---|---|---|---|---|
| **Implementation** | 10 | Who generated the random allocation sequence, who enrolled participants, and who assigned participants to interventions | Replace by 10a, 10b and 10c | 11 |
| | 10a | | Who generated the random allocation sequence, who enrolled clusters, and who assigned clusters to interventions | 11 |
| | 10b | | Mechanism by which individual participants were included in clusters for the purposes of the trial (such as complete enumeration, random sampling) | 7 |
| | 10c | | From whom consent was sought (representatives of the cluster, or individual cluster members, or both), and whether consent was sought before or after randomisation | 7 |
| | | | | |
| **Blinding** | 11a | If done, who was blinded after assignment to interventions (for example, participants, care providers, those assessing outcomes) and how | | **11** |
| | 11b | If relevant, description of the similarity of interventions | | **9** |
| **Statistical methods** | 12a | Statistical methods used to compare groups for primary and secondary outcomes | How clustering was taken into account | 11-12 |
| | 12b | Methods for additional analyses, such as subgroup analyses and adjusted analyses | | **11-12** |
| **Results** | | | | |

| | | | | |
|---|---|---|---|---|
| **Participant flow (a diagram is strongly recommended)** | 13a | For each group, the numbers of participants who were randomly assigned, received intended treatment, and were analysed for the primary outcome | For each group, the numbers of clusters that were randomly assigned, received intended treatment, and were analysed for the primary outcome | 12 |
| | 13b | For each group, losses and exclusions after randomisation, together with reasons | For each group, losses and exclusions for both clusters and individual cluster members | 12 |
| **Recruitment** | 14a | Dates defining the periods of recruitment and follow-up | | **12** |
| | 14b | Why the trial ended or was stopped | | **N/A** |
| **Baseline data** | 15 | A table showing baseline demographic and clinical characteristics for each group | Baseline characteristics for the individual and cluster levels as applicable for each group | Table 1 |
| **Numbers analysed** | 16 | For each group, number of participants (denominator) included in each analysis and whether the analysis was by original assigned groups | For each group, number of clusters included in each analysis | Table 2 |
| **Outcomes and estimation** | 17a | For each primary and secondary outcome, results for each group, and the estimated effect size and its precision (such as 95% confidence interval) | Results at the individual or cluster level as applicable and a coefficient of intracluster correlation (ICC or k) for each primary outcome | Table 2 |
| | 17b | For binary outcomes, presentation of both absolute and relative effect sizes is recommended | | **Table 2** |
| **Ancillary analyses** | 18 | Results of any other analyses | | **Table 2** |

| | | | | |
|---|---|---|---|---|
| | | performed, including subgroup analyses and adjusted analyses, distinguishing pre-specified from exploratory | | |
| **Harms** | 19 | All important harms or unintended effects in each group (for specific guidance see CONSORT for harms[iii]) | | **14** |
| **Discussion** | | | | |
| **Limitations** | 20 | Trial limitations, addressing sources of potential bias, imprecision, and, if relevant, multiplicity of analyses | | **16-17** |
| **Generalisability** | 21 | Generalisability (external validity, applicability) of the trial findings | Generalisability to clusters and/or individual participants (as relevant) | 14-17 |
| **Interpretation** | 22 | Interpretation consistent with results, balancing benefits and harms, and considering other relevant evidence | | **14-17** |
| **Other information** | | | | |
| **Registration** | 23 | Registration number and name of trial registry | | **4** |
| **Protocol** | 24 | Where the full trial protocol can be accessed, if available | | **10.1371/journal.pone.0262237** |
| **Funding** | 25 | Sources of funding and other support (such as supply of drugs), role of funders | | Wellcome Trust & National Institute for Health Research, UK; page 2 |

# APPENDIX: Methods 2: Costing methods

**Overview**

Within the FISH study, we conducted a cost analysis and economic evaluation to determine the costs and assess whether the two interventions: PE and PDE are cost-effective, in comparison to the eSoc arm in Mangochi, Malawi. A provider perspective was used for this work.

A cost-effectiveness analysis was done to estimate the incremental cost per the following trial outcomes: number of fishermen without active schistosomiasis cases or treated, number of fishermen tested for HIV, number of fishermen initiated on ART or VMMC. All costs were expressed in 2022 US dollars. The time horizon for our analysis was 12 months. Due to the main study and time horizon not being more than 1 year, costs and health outcomes were not discounted. Details of the main study's procedures have been reported in the main text and study protocol.

**Healthcare resource use and costs**

Healthcare resource use by participants were collected at the first beach clinic visit and at day 28 (at follow up). During the first visit the following was collected: receipt of presumptive schistosomiasis treatment (praziquantel), oral HIV self-test kits, serial finger-prick HIV testing, referrals made to ART initiation and continuation of ART, and VMMC booking. At day 28, schistosomiasis testing and presence of active schistosomiasis was recorded.

The identification of inputs used for implementing the beach clinic's services and peer led interventions were obtained through semi structured interviews with key research staff, including those working at the beach clinic (study coordinator, laboratory technician at each clinic, and HTC counsellor at each clinic). Interviews were also conducted with one peer leader per arm. The list was further validated with the study team.

Unit costs of the resources used were obtained from the trial's expenditure documents, consultations with key research and procurement staff of which the study procured resources from. We used international medical prices for the unit cost of medication (praziquental) (Frye,2016).

<u>Activities and materials included in the total costs</u>

The total cost for providing HIV services at the beach clinic comprised of the following: staff time (HTC counsellor and lab technician), cost of conducting unigold and determine HIV tests including the equipment and consumables required, and HIV self-test kits (for intervention arms only). As for VMMC linkage, the costs primarily comprised of personnel costs.

The total cost for providing schistosomiasis related services included: staff time (laboratory technician), the cost of praziquental, and the cost of the equipment and consumables required to conduct schistosomiasis testing.

Overhead costs for the beach clinic such as utility bills, communication costs and cleaning services were apportioned to each beach clinic service equally for simplicity. This was also done for shared capital and other costs such as the beach clinic tents, furniture and shared equipment and consumables across services and the cost of training. Equipment including furniture and infrastructure at the beach clinic were annuitized over their useful life with annual discount rate of 3.5%. With regards to the amount of staff time spent on each activity, the staff were asked during the interviews on how much time they spent conducting each service per day. The respective salaries were then apportioned to each service according to the time spent on each service.

The cost of the peer leaders or the intervention included: the cost of producing the leaflets, the compensation given to the peer leaders per day, and the cost of training. The time spent by the peer leaders on explaining each service was solicited and the cost was apportioned accordingly (for the PE and PDE arms where multiple activities were carried out). The cost of the leaflets and training were divided equally across each beach clinic service. HIVST costs were included in the cost of the peer leaders in the PDE as these were distributed by the peer leaders and was not part of the beach clinic service.

All costs were expressed in 2022 US Dollars. For the costs incurred and obtained from years other than the year 2022, these costs were adjusted using the World Bank GDP deflators (World Bank, 2023). All research specific costs were excluded from this analysis.

## APPENDIX: Table 1: Unit costs of healthcare resource use items

| **Healthcare resource** | | **Unit cost (USD)** | **Source** |
|---|---|---|---|
| HIV testing | HIV counsellor time | 1 ·80 | Staff interviews |
| | Determine test | 2·28 | Trial expenditure documents and staff interviews |
| | Unigold test (if positive determine test) | 1·94 | Trial expenditure documents and staff interviews |
| | Consumables used across all tests | 68·42 | Trial expenditure documents and staff interviews |
| **ART & VMMC linkage | HIV counsellors time | 1·35 | Staff interviews |
| Schistosomiasis treatment | Lab technician's time | 0·26 | Primary costing |
| | Praziquantel | 0·44 | International medical products price guide (Fyre 2016) |

| | | | |
|---|---|---|---|
| | Consumables | 0·66 | Trial expenditure documents and staff interviews |
| Schistosomiasis testing | Lab technician's time | 1·04 | Staff interviews |
| | Consumables | 5·60 | Trial expenditure documents and staff interviews |
| | Equipment | 1,674·99 | Trial expenditure documents and staff interviews |
| Shared beach clinic costs | *Capital and equipment | 5,278·08 | Trial expenditure documents and staff interviews |
| | consumables | 95·70 | Trial expenditure documents and staff interviews |
| | Beach clinic staff training | 363·20 | trial expenditure documents |
| | Overhead costs | 253·82 | Trial expenditure documents |
| Intervention costs | Peer leaders | 2·44 | staff interviews |
| | Training (per beach clinic) | 65·76 | Trial expenditure documents |
| | leaflets | 0·03 | Trial expenditure documents |
| | HIV Self-test kits | 3·39 | Trial expenditure documents |

*Capital and equipment costs are given at current purchase price, before annualization.

**Consumables used for this activity only included stationary. As stationary was a shared beach clinic cost, it has been included under the shared beach clinic costs of consumables.

USD: United States Dollar

USD$1·00 = MWK1,026·40 as of 31/12/2022

**Health outcomes**

Health outcomes for the economic evaluation were: number of fishermen without active schistosomiasis or treated for schistosomiasis, number of fishermen tested for HIV, and number of fishermen initiated on ART or VMMC. These outcomes were measured at the first visit to the clinic and at follow up, day 28.

**Cost-effectiveness analysis**

A cost-effectiveness analysis was conducted from the healthcare provider's perspective. This was done by calculating the incremental cost-effectiveness ratio (ICER). ICERs were therefore calculated

by dividing the incremental total costs per arm by the incremental health outcomes observed in the trial. The following health outcomes were used: Number without or treated for schistosomiasis, number of HIV tests conducted, and number of participants linked to VMMC or ART. The primary outcome of the trial is the proportion of active schistosomiasis cases, however for the costing work we have used the number treated for schistosomiasis. This will give the incremental cost to treat or avert an additional schistosomiasis case. To account for the possibility that incremental costs and outcomes may reflect differences in cohort sizes within the study, we calculated the ICERs using a hypothetical cohort size of 2000 for each arm. The relative effect of each intervention found within the study was multiplied by 2000 to get the total numbers per health outcome. For costs, we calculated the total costs for each arm by multiplying arm-specific per person costs by 2000. There is no formal cost-effectiveness threshold for Malawi, regarding this study's outcomes. To the best of our knowledge there has not been similar trials with economic evaluations conducted within a similar population. Therefore, this study will only report the ICERs without relation to a willingness to pay threshold.

**Sensitivity analysis**

We conducted a probabilistic sensitivity analysis by varying the total cost per arm and the study's outcomes (proportion of fishermen tested for HIV, proportion linked to ART/VMMC, and proportion treated for schistosomiasis). The total costs per arm were varied within their respective gamma distributions, and the outcomes were varied within their respective beta distributions, as estimated from the study's results. This was done by simultaneously drawing 10,000 random samples of costs and outcomes from their distributions to estimate incremental costs and outcomes. The eSOC arm was used as the comparator for this analysis. We then plotted the incremental costs against the incremental outcomes (Figure 2).

**References**

Frye, J. E. (2016). *Guide des Prix Internationaux des Produits Médicaux Guía Internacional de Precios de Productos Médicos 2015 International Medical Products Price Guide*. www.msh.org

World Bank. (2023, July 22). *https://data.worldbank.org/indicator/NY.GDP.DEFL.KD.ZG?locations=MW*.

# APPENDIX: Figure 1: Timing of trial activities and follow up

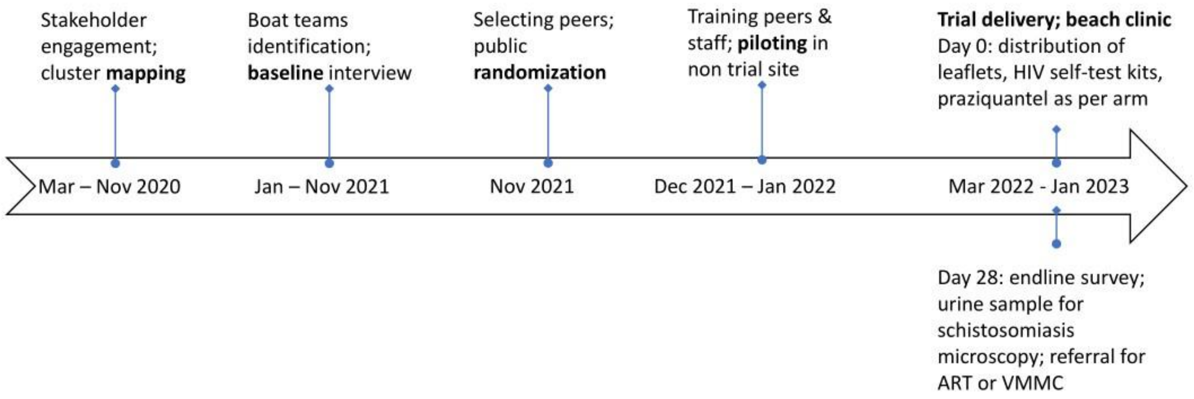


# APPENDIX: Table 2: Total costs by trial arm (2022 USD$)

| **Service** | **eSOC** | **PE** | **PDE** |
|---|---|---|---|
| HIV testing | 7922·45 | 7577·70 | 6508·30 |
| ART/VMMC linkage | 77·16 | 82·94 | 126·17 |
| Schistosomiasis testing | 12227·81 | 11821·39 | 12438·03 |
| Schistosomiasis treatment | 7324·41 | 7306·89 | 7626·22 |
| All services | 27864·62 | 27158·73 | 26944·03 |

USD$1.00 = MWK1,026.40 as of 31/12/2022

# APPENDIX: Table 3: Cost per fisherman by trial arm (2022 USD$)

| | **eSOC** | **PE** | **PDE** |
|---|---|---|---|
| Per HIV test | 5·63 | 5·76 | 4·39 |
| Per ART/VMMC linkage | 1·70 | 1·61 | 1·73 |
| Per fisherman put on schistosomiasis treatment | 4·20 | 4·33 | 4·30 |
| Per fisherman treated for schistosomiasis | 5·04 | 5·13 | 4·97 |
| Total cost per fisherman | 15·97 | 16·10 | 15·18 |

USD$1·00 = MWK1,026·40 as of 31/12/2022

As shown in Appendix: Table 4, treating schistosomiasis costs more and is more effective under the PE arm. The incremental cost per additional fisherman treated for schistosomiasis in the PE arm was $11.42 as compared to the eSOC arm. Under the PDE arm, treating schistosomiasis is less costly, and more effective as compared to

the eSOC arm, therefore resulting to the PDE arm being dominant.  Due to the PE arm being more costly and less effective in testing for HIV, the PE arm was dominated by the eSOC arm. However, the PDE arm dominates the eSOC arm as testing for HIV costs less and is more effective as compared to the eSOC arm. ART/VMMC linkage delivered under the PE intervention arm is more costly but more effective than the eSOC arm. Under the PDE arm, ART/VMMC linkage is less costly and less effective as compared to the eSOC arm. The incremental cost per additional fisherman linked to ART/VMMC was -$3.76 and $73.84 in the PE and PDE arms, respectively.

We further compared the PDE arm to the PE arm (Appendix: table 4). The results demonstrated that with regards to schistosomiasis treatment and HIV testing the PDE arm dominates the PE arm. With regards to ART/VMMC linkage, the PDE arm was found to be less costly and less effective as compared to the PE arm.

Figure 2 shows the results of the probabilistic sensitivity analysis. The three graphs plot the incremental total cost of each intervention arm against the incremental outcomes of the study. Therefore, graphs A, B, and C show the incremental total cost of each intervention against the incremental proportion of fishermen tested for HIV, the incremental proportion of fishermen linked to ART/VMMC, and the incremental proportion of fishermen treated for schistosomiasis. For this analysis the eSOC arm was the comparator. Graph A demonstrates that HIV testing under the PE arm is less effective, with little difference in costs compared to the eSOC arm. Under the PDE arm, conducting HIV testing was more effective whilst slightly being less costly. Demonstrating that most estimates under the PDE arm showed dominance over the eSOC arm. Graph B shows that under the PE arm linkage to ART/VMMC is slightly more costly whilst being more effective as compared to the eSOC arm. ART/VMMC linkage under the PDE arm is shown to be less costly whilst being less effective. Shown in graph C, the PE arm is shown to be more effective in treating schistosomiasis whilst being minimally more costly than the eSOC arm. Treating schistosomiasis under the PDE arm is more effective and less costly than the eSOC arm. The majority of estimates demonstrated that the PDE arm dominates the eSOC arm with regards to treating schistosomiasis.

## APPENDIX: Table 4: Incremental cost effectiveness ratios

| **Trial outcome** | **SOC**<br><br>**N=2000** | **PE**<br><br>**N=2000** | **PDE**<br><br>**N=2000** |
|---|---|---|---|
| **Total cost (all services)** | **31936·50** | **32197·70** | **30359·50** |

| | | | | |
|---|---|---|---|---|
| **Incremental cost** | | **-** | **261·13** | **-1577·06** |
| Tested for HIV | N (%) | 1611 (85·2%) | 1559 (84·4%) | 1671 (88·0%) |
| | Incremental outcome | - | -52·48 | 59·52 |
| | ICER | - | Dominated | Dominant |
| Linked to ART/VMMC | N (%) | 264 (13·2%) | 333 (16·7%) | 242 (12·1%) |
| | Incremental outcome | - | 69·53 | -21·36 |
| | ICER | - | 3·76 | 73·84 |
| Treated for schistosomiasis | N (%) | 1665 (83·3%) | 1688 (84·4%) | 1728 (86·4%) |
| | Incremental outcome | - | 22·87 | 63·12 |
| | ICER | - | 11·42 | Dominant |
| **Incremental cost (PDE compared to PE)** | | | **-** | **-1838·20** |
| Tested for HIV | Incremental outcome | | - | 112 |
| | ICER | | - | Dominant |
| Linked to ART/VMMC | Incremental outcome | | - | -91 |
| | ICER | | - | 20·2 |
| Treated for schistosomiasis | Incremental outcome | | - | 40 |
| | ICER | | - | Dominant |

USD$1·00 = MWK1,026·40 as of 31/12/2022
ICER: Incremental cost-effectiveness ratio
Figures calculated using a hypothetical cohort of 2000 participants per arm

# APPENDIX: Figure 2: Cost-effectiveness scatter plots for sensitivity analysis

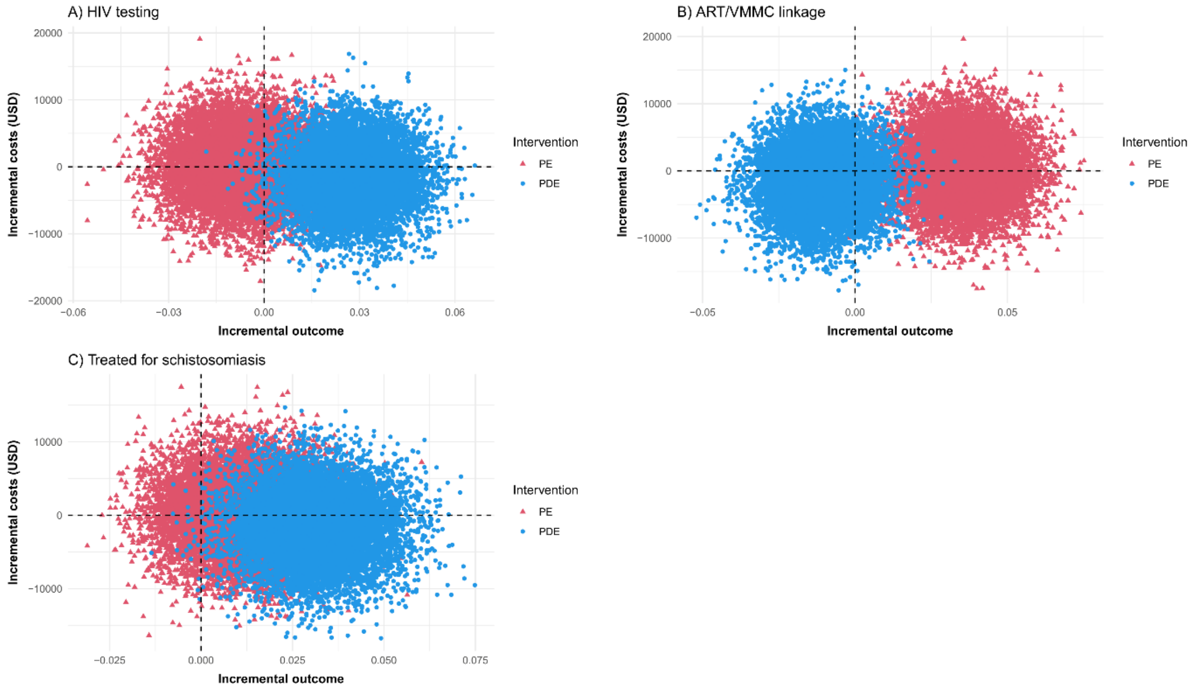


USD$1·00 = MWK1,026·40 as of 31/12/2022

A) Plots the incremental total costs against the incremental proportion of fishermen tested for HIV.
B) Plots the incremental total costs against the incremental proportion of fishermen linked to ART or VMMC.
C) Plots the incremental total costs against the incremental proportion of fishermen treated for schistosomiasis.

Comparisons were made to the eSOC arm.

# APPENDIX: Table 5: Primary and secondary outcomes under complete case (sensitivity analysis)

| | eSOC arm (clusters = 15; N = 1519) | PE arm (clusters = 15; N = 1450) | PDE arm (clusters = 15; N = 1560) |
|---|---|---|---|
| Primary outcome 1: Fishermen with active Schistosomiasis† (%) | 218 (14·4%) | 212 (14·6%) | 196 (12·6%) |
| Risk difference (95% CI) % | | 0·27 (-2·27; 2·81) | -1·81 (-0·04; 0·01) |
| Unadjusted RR (95% CI); p-value | 1 | 1·02 (0·86; 1·21); p = 0·833 | 0·87 (0·73; 1·05); p = 0·143 |
| Adjusted RR** (95% CI); p-value | 1 | 1·01 (0·85; 1·21); p = 0·885 | 0·87 (0·72; 1·04); p=0·116 |
| Primary outcome 2: Fishermen initiated ART or booked for VMMC* (%) during study period; excludes those already on ART | 161 (10·6%) | 219 (15·1%) | 168 (10·8%) |
| Risk difference (95% CI) % | | 4·39 (1·95; 6·83) | 0·12 (-2·11; 2·36) |
| Unadjusted RR (95% CI); p-value | 1 | 1·41 (1·16; 1·71); p < 0·0001 | 1·01 (0·82; 1·24); p = 0·914 |
| Adjusted RR** (95% CI); p-value | 1 | 1·32 (1·09; 1·59); p = 0·005 | 0·97 (0·79; 1·20); p = 0·809 |

| | | | |
|---|---|---|---|
| Secondary outcome: Fishermen tested for HIV‡ (%), excludes those already on ART | 1212/1437 (84·3%) | 1094/1340 (81·6%) | 1306/1482 (88·1%) |
| Risk difference (95% CI) % | | -2·70 (-5·50; 0·00) | 3·78 (1·28; 6·28) |
| Unadjusted RR (95% CI); p-value | 1 | 0·97 (0·94; 1·00); p = 0·058 | 1·04 (1·01; 1·08); p = 0·003 |
| Adjusted RR** (95% CI); p-value | 1 | 0·98 (0·94; 1·02); p = 0·085 | 1·03 (1·01; 1·03); p = 0·006 |
| Secondary outcome: Fishermen perceived acceptability of pre-exposure prophylaxis (%) | 808/1294 (62·4%) | 926/1224 (75·7%) | 882/1309 (67·4%) |
| Risk difference | | 12·97 (9·33; 16·62) | 4·71 (0·97; 8·44) |
| Unadjusted RR (95% CI); p-value | 1 | 1.21 (1·15; 1·28); p < 0·0001 | 1·08(1·02;1·14); p = 0·008 |
| Adjusted RR** (95% CI); p-value | 1 | 1.21 (1·15; 1·28); p < 0·0001 | 1·08 (1·02; 1·15); p = 0·006 |
| Secondary outcome: Self-reported high-risk sex in the last 1 month (%) | 94/1517 (6·2%) | 124/1448 (8·6%) | 69/1558 (4·4%) |
| | | 2·37 (0·48; 4·25) | -1·18 (-3·35; -0·18) |
| Unadjusted RR (95% CI); p-value | 1 | 1.38 (1·07; 1·79); p = 0·014 | 0·71(0·53;0·97); p = 0·030 |
| Adjusted RR** (95% CI); p-value | 1 | 1.28 (1·00; 1·65); p = 0·054 | 0·74 (0·55; 0·99); p = 0·046 |
| Secondary outcome: Self-reported schistosomiasis knowledge (%) | 1272/1387 (91·7%) | 1305/1407 (92·8%) | 1298/1450 (89·5%) |
| | | 1·05 (-0·96; 3·06) | -2·19 (-4·37; -0·02) |
| Unadjusted RR (95% CI); p-value | 1 | 1.01 (0·99; 1·03); p = 0·339 | 0·98 (0·95;1·00); p = 0·045 |
| Adjusted RR** (95% CI); p-value | 1 | 1.00 (0·98; 1·02); p = 0·833 | 0·98 (0·95; 1·00); p = 0·026 |

eSOC: enhanced standard of care; PE: peer educators; PDE: peer distributor educator; RR: risk ratio; CI: confidence interval; ART: antiretroviral therapy; VMMC: voluntary male medical circumcision. † Measured within 28 days, 2) having ≥ 1 S. haematobium egg seen on light microscopy of the filtrate from 10mls urine (“egg-positive”). ** Adjusted for clustering, literacy and HIV testing in last 12 months and VMMC status. Intra cluster correlation coefficient (ICC) = 0·08, estimated from the adjusted model

# APPENDIX: Table 6: Primary and secondary outcomes results using logistic regression

| | eSOC arm (clusters = 15; N = 1745) | PE arm (clusters = 15; N = 1687) | PDE arm (clusters = 15; N = 1775) |
|---|---|---|---|
| Primary outcomes | | | |
| Fishermen with active Schistosomiasis† (%) | 292 (16·7%) | 263 (15·6%) | 241 (13·6%) |
| Unadjusted OR (95% CI); p-value | 1 | 0·92 (0·77; 1·10); p = 0·367 | 0·78 (0·65; 0·94); p = 0·009 |
| Adjusted OR** (95% CI); p-value | 1 | 0·89 (0·74; 1·07); p = 0·220 | 0·78 (0·65; 0·94); p=0·008 |
| Fishermen initiated ART or scheduled for VMMC* (%) during study period; excludes those already on ART | 230 (13·2%) | 281 (16·7%) | 215 (12·1%) |
| Unadjusted OR (95% CI); p-value | 1 | 1·32 (1·09; 1·59); p = 0·004 | 0·91 (0·74; 1·11); p = 0·341 |
| Adjusted OR** (95% CI); p-value | 1 | 1·19 (0·97; 1·46); p = 0·087 | 0·86 (0·74; 1·05); p = 0·171 |
| Fishermen tested for HIV‡ (%), excludes those already on ART | 1406/1650 (85·2%) | 1315/1558 (84·4%) | 1483/1686 (88·0%) |
| Unadjusted OR (95% CI); p-value | 1 | 1·02 (0·67; 1·54); p = 0·927 | 25·44 (7·87; 155·84); p < 0·0001 |
| Adjusted OR** (95% CI); p-value | 1 | 1·00 (0·99; 1·01); p = 0·526 | 1·01 (1·01; 1·02); p = 0·009 |
| Secondary outcomes | | | |
| Fishermen perceived acceptability of pre-exposure prophylaxis (%) | 1130 (64·8%) | 1329 (78·9%) | 1238 (69·8%) |
| Unadjusted OR (95% CI); p-value | 1 | 1.22 (1·17; 1·27); p < 0·0001 | 1·08 (1·03;1·13); p = 0·002 |
| Adjusted OR** (95% CI); p-value | 1 | 1·22 (1·17; 1·27); p < 0·0001 | 1·08 (1·03; 1·13); p < 0·0001 |
| Self-reported high-risk sex in the last 1 month (%) | 96 (5·5%) | 124 (7·4%) | 69 (3·9%) |
| Unadjusted OR (95% CI); p-value | 1 | 1.41 (1·08; 1·86); p = 0·013 | 0·73 (0·54; 1·00); p = 0·054 |
| Adjusted OR** (95% CI); p-value | 1 | 1.40 (1·06; 1·85); p = 0·024 | 0·77 (0·56; 1·05); p = 0·103 |
| Self-reported schistosomiasis knowledge (%) | 1612 (92·4%) | 1568 (93·1%) | 1607 (90·5%) |
| Unadjusted OR (95% CI); p-value | 1 | 1.11 (0·85; 1·43); p = 0·445 | 0·79 (0·61;1·00); p = 0·051 |
| Adjusted OR** (95% CI); p-value | 1 | 1.01 (0·78; 1·32); p = 0·926 | 0·79 (0·62; 1·00); p = 0·054 |

eSOC: enhanced standard of care; PE: peer educators; PDE: peer distributor educator; RR: risk ratio; CI: confidence interval; ART: antiretroviral therapy; VMMC: voluntary male medical circumcision. † Measured within 28 days, 2) having ≥ 1 S. haematobium egg seen on light microscopy of the filtrate from 10mls urine (“egg-positive”). ** Adjusted for clustering, literacy and HIV testing in last 12 months and VMMC status. Intra cluster correlation coefficient (ICC) = 0·08, estimated from the adjusted model

[i] Hopewell S, Clarke M, Moher D, Wager E, Middleton P, Altman DG, et al. CONSORT for reporting randomised trials in journal and conference abstracts. *Lancet* 2008, 371:281-283

[ii] Hopewell S, Clarke M, Moher D, Wager E, Middleton P, Altman DG at al (2008) CONSORT for reporting randomized controlled trials in journal and conference abstracts: explanation and elaboration. *PLoS Med* 5(1): e20

[iii] Ioannidis JP, Evans SJ, Gotzsche PC, O'Neill RT, Altman DG, Schulz K, Moher D. Better reporting of harms in randomized trials: an extension of the CONSORT statement. *Ann Intern Med* 2004; 141(10):781-788.
