# Supplementary material for: Combined interventions for the testing and treatment of HIV and schistosomiasis among fishermen in Malawi: a three-arm, cluster-randomised trial
Source: Lancet Glob Health. 2024 Sep 18;12(10):e1673–83. doi: 10.1016/S2214-109X(24)00283-3 (PMC11420466; doi:10.1016/S2214-109X(24)00283-3)
Supplement: Equitable Partnership Declaration [file mmc2.pdf]

THE LANCET
Global Health

Supplementary appendix 2

This Equitable Partnership Declaration (EPD) was submitted by the authors, and we reproduce it as supplied. It has not been peer reviewed. *The Lancet*'s editorial processes have not been applied to the EPD.

Supplement to: Choko AT, Dovel KL, Kayuni S, et al. Combined interventions for the testing and treatment of HIV and schistosomiasis among fishermen in Malawi: a three-arm, cluster-randomised trial. *Lancet Glob Health* 2024; **12:** e1673–83.

<u>Equitable Partnership Declaration questions</u>

<u>Researcher considerations</u>

1. Please detail the involvement that researchers who are based in the region(s) of study had during a) study design; b) clinical study processes, such as processing blood samples, prescribing medication, or patient recruitment; c) data interpretation; and d) manuscript preparation, commenting on all aspects. If they were not involved in any of these aspects, please explain why.

   This question is intended for international partnerships; if all your authors are based in the area of study, this question is not applicable.

   This should include a thorough description of their leadership role(s) in the study. Are local researchers named in the author list or the acknowledgements, or are they not mentioned at all (and, if not, why)? Please also describe the involvement of early career researchers based in the location of the study. Some of this information might be repeated from the Contributors section in the manuscript. Note: we adhere to ICMJE authorship criteria when deciding who should be named on a paper.

**a) Study design:** The first author (ATC) is a Malawian who lives and works in Malawi who led the design of the study together with ELC.
**b) Clinical study processes:** ATC and SK, MMT, all from Malawi were responsible for the collection and processing of urine samples for schistosomiasis diagnosis, and HIV testing for participants.
**c) Data interpretation:** ATC, JJ, and MMT all Malawians were responsible for the interpretation of the epidemioogial aspects of the study. WK a Malawian was responsible for the interpretation of the health economics aspects of the study.
**d) Manuscript preparation:** ATC, a Malawian, led the drafting, revision and submission of the manuscript.

2. Were the data used in your study collected by authors named on the paper, or have they been extracted from a source such as a national survey? ie, is this a secondary analysis of data that were not collected by the authors of this paper. If the authors of this paper were not involved in data collection, how were data interpreted with sufficient contextual knowledge?

   The Lancet Global Health *believe contextual understanding is crucial for informed data analysis and interpretation.*

Data used in the paper were collected by the authors named on the paper.

3. How was funding used to remunerate and enhance the skills of researchers and institutions based in the area(s) of study? And how was funding used to improve research infrastructure in the area of study?

   Potentially effective investments into long-term skills and opportunities within institutions could include training or mentorship in analytical techniques and manuscript writing, opportunities to lead all or specific aspects of the study, financial remuneration rather than requiring volunteers, and other professional development and educational opportunities.

   Improvements to research infrastructure could be funding of extended trial designs (such as platform trials) and use of master protocols to enable these designs, establishment of long-term contracts for research staff, building research facilities, and local control of funding allocation.

**Skills:**<br>Malawians: ATC, SK and MMT enhanced their epidemiological skills especially on the design and conduct of cluster randomized trials. JJ enhanced his statistical skills especially on handling clustered data requiring multiple imputation. WK used the health economics data generated in study to enhance her skills in computing unit costs and how to conduct cost-effectiveness analysis.
**Research infrastructure:**<br>The Malawi Liverpool Wellome Programme built mobile infrastructure to handle mobile research units in logistically challenging environments. This was through the use of mobile beach clinics mounted on the lakeshore to support HIV and schistosomiasis services.

4. How did you safeguard the researchers who implemented the study?

   Please describe how you guaranteed safe working conditions for study staff, including provision of appropriate personal protective equipment, protection from violence, and prevention of overworking.

The study followed health and safety procedures laid down by the Malawi Liverpool Wellcome Programme. Staff working in beach clinics were given safe drinking water, free housing and time off as needed to facilitate a safe working environment.

<u>Benefits to the communities and regions of study</u>

5. How does the study address the research and policy priorities of its location?

   How were the local priorities determined and then used to inform the research question? Who decided which priorities to take forward? Which elements of the study address those priorities?

We engaged the Ministry of Health (HIV/AIDS Department) and the Schistosomiasis Control Programme before, during and after study implementation. Both HIV and schistosomiasis diagnosis and treatment are national policies and the work aimed to find optimal models of ensuring that services reach the fishermen as desired by the Ministry of Health.

6. How will research products be shared in the community of study?

   For instance, will you be providing written or oral layperson summaries for non-academic information sharing? Will study data be made available to institutions in the region(s) of study? The Lancet Global Health *encourages authors to translate the summary (abstract) into relevant languages after paper editing; do you intend to translate your summary?*

   We organized end of study community dissemination meetings with the local health facilities, the fishermen and community leaders.

7. How were individuals, communities, and environments protected from harm?

a) *How did you ensure that sensitive patient data was handled safely and respectfully? Was there any potential for stigma or discrimination against participants arising from any of the procedures or outcomes of the study?*

   There was potential for stigma from unintended disclosure of HIV or schistosomiasis status of the participants in the study. We followed Good Clinical Practice in all study procedures and ensured that all HIV testing components did not identify participants by name but rather through an ID. The HIV services were offered in a status neutral approach where both HIV positive and HIV negative individuals were all mixed to the extent it was not possible to work out procedures by HIV status. Participants were informed that the peers distributing HIV self-test kits did not need to know the results of the testing.

b) *Might any of the tests be experienced as invasive or culturally insensitive?*

   N/A – all procedures were standard.

c) *How did you determine that work was sensitive to traditions, restrictions, and considerations of all cultural and religious groups in the study population?*

   We translated the study leaflets, information sheets and consent forms into the local language (Chichewa) to ensure that they were accessible to the majority of the study participants. The field team was recruited from within the local area while ensuring anonymity to ensure that they would use the local language to engage with the participants.

d) *Were biowaste and radioactive waste disposed of in accordance with local laws?*

   All biowastes such as for urine-based testing and HIV testing were incinerated in accordance with the safety and environmental procedures laid down by MLW.

e) *Were any structures built that would have impacted members of the community or the environment (such as handwashing facilities in a public space)? If so, how did you ensure that you had appropriate community buy-in?*

   We provided handwashing facilities at the beach clinic and provided face masks during the COVID-19 time to ensure safety of our staff and that of study participants.

f) *How might the study have impacted existing health-care resources (such as staff workloads, use of equipment that is typically employed elsewhere, or reallocation of public funds)?*

We referred fishermen who tested HIV positive or those who tested HIV negative and wanted circumcision to the nearest ART clinic. This may have placed further demand for the health facilities but we beefed up staff members where necessary and we did not experience demand that placed too extraneous needs on existing staff. In fact, the circumcision procedure is done by batching and so the health facilities was grateful for our demand creation activities.

8. Finally, please provide the title (eg, Dr/Prof, Mr/Mrs/Ms/Mx), name, and email address of an author who can be contacted about this statement. This can be the corresponding author.

**Name:** Augustine T. Choko<br>**Email: achoko@mlw.mw**
